# Alternative Factors in Possible Involvement of Coronary Microvascular Dysfunction in Older Patients with HFpEF

**DOI:** 10.3390/jcm13195911

**Published:** 2024-10-03

**Authors:** Shiro Hoshida, Tetsuya Watanabe, Nobutaka Masunaga, Yukinori Shinoda, Masahiro Seo, Takaharu Hayashi, Masamichi Yano, Takahisa Yamada, Yoshio Yasumura, Shungo Hikoso, Katsuki Okada, Daisaku Nakatani, Yohei Sotomi, Yasushi Sakata

**Affiliations:** 1 Department of Cardiovascular Medicine, Yao Municipal Hospital, 1-3-1 Ryuge-cho, Yao 581-0069, Japan; t.watanabe252@gmail.com (T.W.); nmas1103@yahoo.co.jp (N.M.); yshinoda1031@gmail.com (Y.S.); 2 Division of Cardiology, Osaka General Medical Center, Osaka 558-8558, Japan; roland-dyens@hotmail.co.jp (M.S.); takaymda@nike.eonet.ne.jp (T.Y.); 3 Cardiovascular Division, Osaka Police Hospital, Osaka 543-0035, Japan; takaharu0866@yahoo.co.jp; 4 Division of Cardiology, Osaka Rosai Hospital, Sakai 591-8025, Japan; myano0820@gmail.com; 5 Division of Cardiology, Amagasaki-Chuo Hospital, Amagasaki 661-0976, Japan; yasumu@chuoukai.or.jp; 6 Department of Cardiovascular Medicine, Osaka University Graduate School of Medicine, Suita 565-0871, Japan; hikoso@cardiology.med.osaka-u.ac.jp (S.H.); okada.katsuki.med@osaka-u.ac.jp (K.O.); nakatani@cardiology.med.osaka-u.ac.jp (D.N.); yohei.sotomi@cardiology.med.osaka-u.ac.jp (Y.S.); yasushisk@cardiology.med.osaka-u.ac.jp (Y.S.); 7 Department of Cardiovascular Medicine, Nara Medical University, Kashihara 634-8522, Japan; 8 Department of Medical Informatics, Osaka University Graduate School of Medicine, Suita 565-0871, Japan

**Keywords:** atrial fibrillation, coronary microvascular dysfunction, diastolic blood pressure, heart failure, left ventricular hypertrophy

## Abstract

**Objectives**: Coronary microvascular dysfunction (CMD) is associated with many heart diseases, including heart failure (HF) with preserved ejection fraction (HFpEF). Invasive examinations for CMD detection are difficult in older patients with HFpEF, and the decision criteria for noninvasive CMD measurements are unclear. We aimed to identify alternative factors in the possible involvement of CMD in the progression and prognosis of HFpEF. **Methods**: We analyzed 607 patients with HFpEF who were hospitalized for acute decompensated HF without a history of coronary artery disease (CAD). Blood tests and transthoracic echocardiography were performed. We focused on left ventricular hypertrophy (LVH) and coronary perfusion pressure (diastolic blood pressure, dBP). **Results**: The patients with LVH showed reduced diastolic function (E/e’) and a lower incidence of atrial fibrillation (AF) compared with those without LVH, with no differences in age or dBP. No differences were observed in all-cause mortality between patients with low and high dBP without LVH. In the patients with LVH, the incidence of all-cause mortality was significantly higher, with a lower incidence of AF, reduced renal function, and higher C-reactive protein levels in those with low dBP than in those with high dBP. The comprehensive diastolic functional index, diastolic elastance/arterial elastance, was markedly higher in the patients with LVH, especially in those with all-cause mortality. This index, but not E/e’, was a significant prognostic index in the multivariate Cox hazard analysis when adjusting for age, sex and N-terminal pro-brain natriuretic peptide levels. **Conclusions**: LVH and dBP were clinically important factors in elderly HFpEF patients without a history of CAD.

## 1. Introduction

The new ESC Guidelines on the management of chronic coronary syndromes include a focus on both larger and smaller coronary arteries, and prompt cardiologists to reconsider these syndromes as caused not only by obstruction of large arteries but also by dysfunction of microvascular arteries [1]. Coronary microvascular dysfunction (CMD) is associated with many heart diseases, including heart failure (HF) with preserved ejection fraction (HFpEF) [2,3]. It was found to play an important role as an endotype of HFpEF [4,5,6,7], in addition to its relationship with the outcome of HFpEF [8], although the participants in these studies were relatively young. However, in a significantly aging society, invasive examinations for CMD detection are performed in a small number of patients with HFpEF, because those admitted with HFpEF are markedly old in real-world practice [9]. Furthermore, noninvasive measurements of CMD, such as positron emission tomography [10] and cardiac magnetic resonance [11], are limited to certain hospitals, and the decision criteria for their use in CMD detection are unclear [2]. Therefore, we aimed to detect alternative factors in the possible involvement of CMD in patients with HFpEF, by using general data obtained from older patients admitted with HFpEF. Considering the structural and functional mechanisms of CMD, we focused on left ventricular (LV) hypertrophy (LVH) and coronary perfusion pressure (diastolic blood pressure [dBP]) in relation to LV diastolic function and the prognosis of HFpEF. As a comprehensive index of LV diastolic function, we used a novel echocardiographic parameter that is useful for evaluating the prognosis of HFpEF [12,13]: the ratio of LV diastolic elastance (Ed) to arterial elastance (Ea), which is the relative ratio of LV filling pressure to LV end-systolic blood pressure [14].

## 2. Methods

### 2.1. Study Subjects

We enrolled 607 patients (male/female, 250/357; mean age, 81 years (30–101 years)) with prognostic data from the PURSUIT HFpEF registry [15] from between June 2016 and February 2020 at discharge during index hospitalization for acute decompensated HF. Patients were enrolled based on the previously reported Framingham and LV ejection fraction (LVEF) criteria. The PURSUIT HFpEF study has a prospective, multicenter, observational design in the Osaka region of Japan and includes demographic, clinical, and outcome data from patients hospitalized for HFpEF [15,16]. In the present study, we excluded patients with severe aortic stenosis, aortic regurgitation, mitral stenosis, or mitral regurgitation due to structural changes in the valves; those with a history of coronary artery disease (CAD); and those without data regarding LV mass index (LVMI) and diastolic function (E/e’) detected by transthoracic echocardiography (TTE).

### 2.2. Data Collection and Follow-Up/Clinical Outcome

The methods used for data collection and follow-up/clinical outcome determination, including survival data, have been previously reported [15,16]. The primary end point of this study was all-cause mortality. Collaborating hospitals were encouraged to enroll consecutive patients with HFpEF, irrespective of treatment.

### 2.3. Patient Laboratory Data and Echocardiography Examination

Laboratory data and TTE indices were examined before discharge. Blood pressure and heart rate were measured along with echocardiographic examinations, which were recorded according to the American Society of Echocardiography or European Society of Echocardiography guidelines [17,18]. As a comprehensive index of LV diastolic function, we examined vascular-resistance-independent Ed/Ea ((E/e’)/(0.9 × systolic blood pressure))^12^. We focused on LVH (LVMI: male > 115 g/m^2^, female > 95 g/m^2^) and dBP, and the association with LV diastolic function and prognosis.

### 2.4. Patient and Public Involvement

The PURSUIT HFpEF registry was established according to the principles of the Declaration of Helsinki. The study protocol was approved by the ethics committee of each participating hospital (Osaka University Clinical Research Review Committee, R000024414; Yao Municipal Hospital 2016-0006). All participants provided written informed consent regarding the design and conduct of the study during index hospitalization. We only performed usual examinations in routine clinical practice.

### 2.5. Statistical Analysis

Continuous variables are expressed as mean ± standard deviation, whereas categorical variables are presented as frequencies and percentages. Differences in categorical variables between the groups were assessed using the chi-squared test, whereas those for continuous variables were assessed using Student’s *t*-test. Survival curves were estimated using Kaplan–Meier survival analysis, and the groups were compared using a log-rank test. Multivariable Cox proportional hazards regression analysis was performed after adjusting for age, sex, and N-terminal pro-brain natriuretic peptide (NT-proBNP) levels. Statistical significance was set at *p* < 0.05. All statistical analyses were performed using EZR (Saitama Medical Center, Jichi Medical University, Saitama, Japan).

## 3. Results

### 3.1. Clinical and Laboratory Characteristics of Patients with HFpEF with and without LVH

Table 1 shows a comparison of the clinical and laboratory characteristics and medications between patients with (*n* = 284) and without (*n* = 323) LVH for all the patients and for those with all-cause mortality. No differences were observed in age and dBP; however, body mass index (BMI), systolic blood pressure (sBP), the incidence of female sex, hypertension, dyslipidemia, and atrial fibrillation (AF) were significantly different between those with and those without LVH. In the patients with all-cause mortality, a significant difference was observed in dBP in association with differences in BMI and the incidence of female sex and AF between patients with and without LVH.

In terms of the echocardiographic parameters, the left atrial volume index (LAVI), LV volume index, LVMI, and E/e’ values were significantly higher in the patients with LVH than in those without LVH (Table 2). In cases of all-cause mortality, the LAVI, LVMI, and E/e’ values were significantly different between patients with and without LVH. The E-wave deceleration time, E/A, and LVEF did not differ significantly between the two groups.

### 3.2. Differences in Clinical Characteristics of Patients with HFpEF between Low and High dBP

There were significant differences in age, male sex, sBP, and heart rate between the patients with low and high dBP (Table 3). In the patients without LVH, no significant differences were observed in age, incidence of male sex, or AF between those with low and high dBP. However, in the patients with LVH, there were significant differences in age and the incidence of male sex and AF, which were associated with significant differences in the levels of C-reactive protein (CRP) and estimated glomerular filtration rate (eGFR) between those with low and high dBP (Table 3). No significant correlations were observed between LVMI and dBP in any of the patients (r = −0.032, *p* = 0.43). However, there was a significant positive correlation between dBP and hematocrit (Hct) levels in all the patients with HFpEF (r = 0.208, *p* < 0.001), especially in those with LVH (r = 0.276, *p* < 0.001).

Regarding the echocardiographic data, no differences were observed between patients with low and high dBP or those with or without LVH (Supplemental Table S1).

### 3.3. Significance of a Comprehensive Diastolic Index, Ed/Ea, in Patients with HFpEF

The Ed/Ea levels were significantly higher in the patients with LVH than in those without LVH (Table 4). The Ed/Ea levels were also significantly different between the patients with and without all-cause mortality, both for all the patients and for those with and without LVH. This relationship was also observed between patients with and without LVH, and with low or high dBP. Ed/Ea levels were significantly higher in the patients with LVH than in those without LVH, in patients with both low and high dBP. Furthermore, the Ed/Ea levels in the patients with low dBP were markedly higher than those in the patients with high dBP, both with and without LVH. The highest Ed/Ea values were observed in the patients with LVH with all-cause mortality (Table 4).

### 3.4. Prognostic Analysis for All-Cause Mortality

During the mean follow-up period of 660 days, the number of people who died of any cause was 123 (20%, male/female: 51/72) (Table 1). No significant differences were observed in all-cause mortality between the patients with (59/284, 21%) and without (64/323, 20%) LVH (*p* = 0.423). Between the patients with and without all-cause mortality, there were significant differences in age (*p* < 0.001) and BMI (*p* < 0.001) in those without LVH; however, there were significant differences in age (*p* < 0.001) and dBP (*p* = 0.001) in those with LVH (Supplemental Table S2). Only in the patients with LVH were there significant differences in E/e’ and tricuspid regurgitation pressure gradient between those with and without all-cause mortality (Supplemental Table S3).

There was a significant difference in the incidence of all-cause mortality between the patients with low and high dBP (Table 3). In the patients without LVH, no difference was observed in the incidence of all-cause mortality between those with low and high dBP levels. However, in the patients with LVH, there was a significant difference in the incidence of all-cause mortality between the two groups (Table 3).

**Figure 1:**
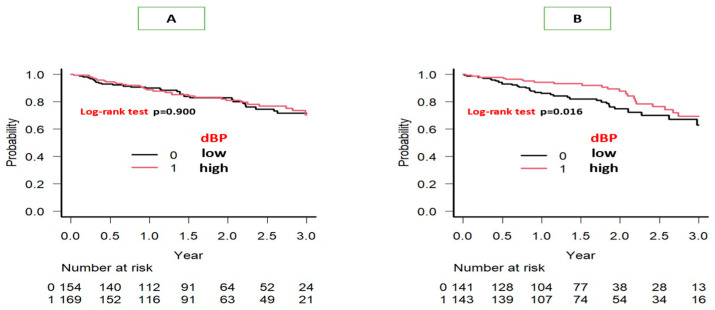
Kaplan–Meier survival curve analysis of all-cause mortality in patients with heart failure with preserved ejection fraction with no left ventricular hypertrophy (LVH) (**A**) and with LVH (**B**). The patients were divided into two groups according to their diastolic blood pressure (dBP) < 65 mmHg (low) or ≥65 mmHg (high) around the examination of transthoracic echocardiography. Low dBP was a significant prognostic factor for all-cause mortality in patients with LVH (Figure 1B, *p* = 0.016).

The dBP was a significant factor for all-cause mortality in the Kaplan–Meier survival curve analysis (log-rank test, *p* = 0.016) in patients with LVH but not in those without LVH (Figure 1). In the univariate Cox hazard analysis, age and levels of albumin, NT-proBNP, and Ed/Ea were significant prognostic factors in patients without LVH. These four factors were also significantly associated with prognosis in the multivariate Cox hazard analysis of these patients (Table 5A). Although the dBP and E/e’ were also significant as prognostic factors associated with the above four factors in the univariable Cox hazard analysis in patients with LVH, only the same four factors were significant in prognosis in the multivariate Cox hazard analysis of the patients with LVH (Table 5B). When E/e’ was used in place of Ed/Ea in the multivariate model for those with LVH, E/e’ was not significant in prognosis (*p* = 0.107).

## 4. Discussion

### 4.1. Involvement of LVH

Chronic coronary syndromes, possibly involving CMD, are a health problem all over the world because a damaged heart caused by malfunctional coronary circulation can cause reduced heart pump function.^1^ Most patients with HFpEF have a history of hypertension resulting in LVH. It is currently believed that patients with LVH generally exhibit CMD, and the degree of CMD is related to the prognosis [19,20]. In this study, patients with HFpEF with LVH showed significant enlargement of the left atrium and ventricle, reduced LV diastolic function and renal function (eGFR), a low incidence of AF and male sex, and a high incidence of hypertension and dyslipidemia compared to those without LVH, although there were no differences in age, dBP, LVEF, or the incidence of all-cause mortality. However, in the patients with all-cause mortality, dBP was significantly lower and BMI was significantly higher in those with LVH than in those without LVH, although no difference was observed in age. Reduced diastolic function has been observed in patients with all-cause mortality, particularly those with LVH. According to these findings, the causes of death may be different between older patients with HFpEF with and without LVH. Large-scale prospective studies are required to investigate differences in the precise causes of death in older patients with HFpEF with and without LVH in real-world practice. However, establishing the causes is quite difficult in older patients.

### 4.2. Involvement of dBP

When the patients were divided into two groups according to dBP, no differences were observed in all-cause mortality between the patients with low and high dBP and those without LVH. In contrast, in the patients with LVH, the incidence of all-cause mortality was significantly higher when associated with older age, reduced renal function, higher CRP levels, and a lower incidence of AF in those with low dBP than in those with high dBP. The mechanisms of CMD are reported to be related to reduced renal function, increased inflammatory response, and AF associated with elevations in E/e’ [2,3,6,21]. According to this evidence, the degree of involvement of renal function, inflammatory reactions, and AF in CMD may differ between patients with LVH with low and high dBP. Renal function and inflammatory reactions may be related to CMD in patients with low dBP. Cardiac microvascular endothelial function is impaired by inflammation and restored by sodium glucose cotransporter 2 inhibitors (SGLT2is) [22,23]. In the present study, we observed a positive correlation between Hct and dBP. The patients treated with SGLT2i exhibited elevations in Hct levels [24,25]. The use of SGLT2is may be a more appropriate therapy for patients with HFpEF with LVH and low dBP. Patients with HF treated with SGLT2i previously showed a significant reduction in sBP, but not in dBP [26]. In contrast, AF may be associated with CMD, particularly in patients with high dBP. There were no differences in the structure of the left atrium and ventricle, nor in the levels of E/e’ and NT-proBNP, between the patients with LVH with low and high dBP.

### 4.3. Comprehensive Diastolic Function

**Figure 2:**
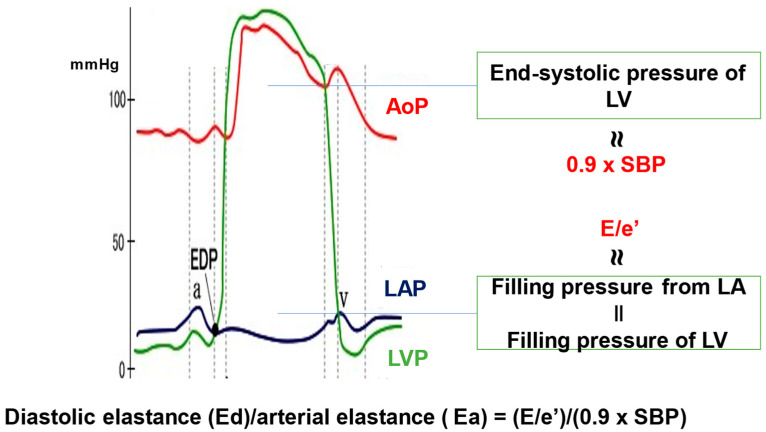
Ratio of left ventricular diastolic elastance (Ed) to arterial elastance (Ea) nearly matches that of filling pressure of left ventricle (filling pressure from left atrium) to end-systolic pressure of left ventricle (modified from [14]). AoP (red), aortic pressure; EDP, end-diastolic pressure; LA, left atrium; LAP (dark blue), left atrial pressure; LV, left ventricle; LVP (green), left ventricular pressure; SBP, systolic blood pressure.

The comprehensive diastolic functional index Ed/Ea = (E/e’)/(0.9 × sBP), was markedly higher in the patients with LVH, especially in those with all-cause mortality. Ed/Ea is an LV diastolic pressure index showing a relative ratio of LV filling pressure (filling pressure from left atrium) to LV end-systolic pressure, but not the slope of LV pressure during the diastolic phase (Figure 2). Ed/Ea, but not E/e’, was a significant prognostic index in the multivariable Cox hazard analysis of these patients. These findings indicate that elderly patients with HFpEF with LVH and low cardiac perfusion pressure exhibit reduced LV diastolic function, which is associated with poor prognosis. In patients with LVH, the hemodynamic state associated with low dBP and without high sBP may be notable in elderly patients with HFpEF. The high dBP observed in young patients with HFpEF may explain secure coronary perfusion pressure, resulting in the mitigation of CMD. Interventions to preserve dBP with no additional load may lead to a new target for improving the prognosis in older patients with HFpEF showing LVH with reduced diastolic function.

### 4.4. Limitations

In elderly patients, all-cause mortality, rather than cardiac death, was examined because the precise determination of cardiac death is challenging, as half of the causes of cardiac death may be sudden death in patients with HF [27]. We did not obtain results regarding the role of LVH and dBP in LV diastolic function and prognosis in young patients with HFpEF, who are typical individuals for interventional studies. We measured the mean value of E/e’ among several beats, because E/e’ can become similar to blood pressure. We also measured blood pressure, along with TTE examination. A large fluctuation in Ed/Ea, the ratio of E/e’ to sBP, does not emerge under stable conditions. We excluded patients with CAD to avoid the possible effects of an obstructed epicardial coronary artery, as in the PROMISE-HFpEF study [5].

## 5. Conclusions

In patients with HFpEF with LVH and low dBP, comprehensive diastolic function was severely depressed in association with poor prognosis compared to those with high dBP, irrespective of NT-proBNP levels. LVH and dBP were clinically important factors, possibly involving CMD, in elderly HFpEF patients without a history of CAD.

## Figures and Tables

**Table 1:** Differences in patient characteristics before discharge between patients with and without LVH in all patients and in those with all-cause mortality.

		All			All-Cause Mortality+		
	All (*n* = 607)	LVH− (*n* = 323)	LVH+ (*n* = 284)	*p*-Value (− vs. +)	LVH− (*n* = 64)	LVH+ (*n* = 59)	*p*-Value (− vs. +)
Age, years	81.2 ± 9.4	81.5 ± 9.2	80.9 ± 9.6	0.425	84.9 ± 7.6	87.0 ± 6.5	0.116
Male, *n* (%)	250 (41)	160 (50)	90 (32)	<0.001	33 (52)	18 (31)	0.014
Body mass index, Kg/m^2^	21.8 ± 4.3	21.3 ± 4.0	22.3 ± 4.5	0.003	19.8 ± 3.6	21.6 ± 4.3	0.012
Systolic blood pressure, mmHg	121 ± 20	119 ± 20	123 ± 19	0.004	115 ± 21	120 ± 20	0.191
Diastolic blood pressure, mmHg	66 ± 12	66 ± 12	66 ± 12	0.855	65 ± 12	61 ± 9	0.038
Heart rate, bpm	70 ± 15	72 ± 15	68 ± 13	<0.001	71 ± 15	71 ± 16	0.961
Albumin, g/dL	3.4 ± 0.4	3.4 ± 0.4	3.4 ± 0.5	0.129	3.2 ± 0.5	3.2 ± 0.4	0.405
CRP, mg/dL	0.78 ± 1.45	0.70 ± 1.27	0.87 ± 1.63	0.136	0.71 ± 1.12	1.17 ± 1.59	0.065
eGFR, mL/min/1.73m^2^	44.2 ± 19.1	47.4 ± 17.3	40.6 ± 19.7	<0.001	44.6 ± 20.5	38.5 ± 23.1	0.130
log (NT-proBNP)	3.02 ± 0.51	2.95 ± 0.46	3.10 ± 0.54	<0.001	3.21 ± 0.42	3.29 ± 0.50	0.333
Atrial fibrillation, *n* (%)	289 (48)	172 (53)	117 (49)	0.001	35 (55)	21 (36)	0.026
Diabetes mellitus, *n* (%)	184 (31)	94 (30)	90 (32)	0.273	18 (29)	22 (37)	0.186
Dyslipidemia, *n* (%)	222 (37)	102 (33)	120 (42)	0.004	14 (23)	19 (32)	0.138
Hypertension, *n* (%)	512 (85)	257 (80)	255 (90)	<0.001	48 (75)	52 (88)	0.051
Medications							
Beta blockers, *n* (%)	315 (52)	161 (50)	154 (54)	0.159	33 (52)	26 (44)	0.257
Calcium channel blockers, *n* (%)	304 (50)	131 (41)	173 (61)	<0.001	28 (44)	29 (49)	0.337
Diuretics, *n* (%)	498 (82)	267 (83)	231 (81)	0.375	54 (84)	54 (92)	0.174
RAAS inhibitors, *n* (%)	435 (72)	234 (72)	201 (71)	0.357	46 (72)	37 (63)	0.186
Statins, *n* (%)	168 (28)	72 (22)	96 (34)	0.001	12 (19)	18 (31)	0.095

Values are mean ± standard deviation or number (%). CRP, C-reactive protein; eGFR, estimated glomerular filtration rate; LVH, left ventricular hypertrophy; NT-proBNP, N-terminal pro-brain natriuretic peptide; RAAS, renin–angiotensin–aldosterone system.

**Table 2:** Differences in echocardiographic data before discharge between patients with and without LVH in all patients and in those with all-cause mortality.

		All			All-Cause Mortality		
	All	LVH−	LVH+	*p*-Value (− vs. +)	LVH−	LVH+	*p*-Value (− vs. +)
LAVI, mL/m^2^	55.1 ± 36.7	49.9 ± 24.7	61.2 ± 35.6	<0.001	54.2 ± 28.5	64.9 ± 27.2	0.049
LVEDVI, mL/m^2^	54.9 ± 20.6	50.0 ± 17.9	60.2 ± 22.0	<0.001	49.6 ± 16.2	57.1 ± 18.6	0.024
LVESVI, mL/m^2^	21.9 ± 10.7	19.9 ± 9.5	24.0 ± 11.6	<0.001	20.0 ± 8.3	22.3 ± 8.8	0.167
LVEF, %	60.8 ± 7.7	60.9 ± 7.9	60.7 ± 7.4	0.836	59.5 ± 6.8	61.4 ± 6.4	0.127
LVMI, g/m^2^	106.1 ± 34.2	83.2 ± 16.5	132.2 ± 30.3	<0.001	82.6 ± 16.5	130.0 ± 25.8	<0.001
TRPG, mmHg	28.4 ± 9.5	28.1 ± 9.2	28.6 ± 9.8	0.510	29.6 ± 9.5	31.7 ± 11.6	0.289
E/A	1.0 ± 0.6	1.0 ± 0.7	1.0 ± 0.5	0.674	1.0 ± 0.6	0.9 ± 0.4	0.671
DcT of E wave	0.21 ± 0.06	0.21 ± 0.06	0.21 ± 0.06	0.288	0.21 ± 0.06	0.22 ± 0.05	0.166
E/e’	13.5 ± 5.7	12.4 ± 5.1	14.7 ± 6.2	<0.001	13.3 ± 4.8	16.6 ± 5.9	0.008

Values are mean ± standard deviation. LAVI, left atrial volume index; LVEDVI, left ventricular end-diastolic volume index; LVESVI, left ventricular end-systolic volume index; LVEF, left ventricular ejection fraction; LVH, left ventricular hypertrophy; LVMI, left ventricular mass index; TRPG, tricuspid regurgitation pressure gradient; DcT, deceleration time; E, early transmitral flow velocity; e’, onset of early diastolic mitral annular velocity.

**Table 3:** Differences in characteristics before discharge between patients with low and high diastolic blood pressure in those with and without LVH.

	Diastolic Blood Pressure		*p*-Value (− vs. +)	LVH− (*n* = 323)		*p*-Value (− vs. +)	LVH+ (*n* = 284)		*p*-Value (− vs. +)
				Diastolic Blood Pressure			Diastolic Blood Pressure		
	Low (*n* = 295)	High (*n* = 312)		Low (*n* = 154)	High (*n* = 169)		Low (*n* = 141)	High (*n* = 143)	
All-cause mortality, *n* (%)	69 (23)	54 (17)	0.039	32 (21)	32 (19)	0.391	37 (26)	22 (15)	0.017
Age, years	82.4 ± 8.1	80.1 ± 10.3	0.001	82.0 ± 8.7	81.1 ± 9.5	0.372	82.9 ± 7.4	78.9 ± 11.0	<0.001
Male, *n* (%)	136 (46)	114 (37)	0.010	84 (55)	76 (45)	0.053	52 (37)	38 (27)	0.041
Body mass index, Kg/m^2^	21.6 ± 4.1	21.9 ± 4.5	0.445	21.0 ± 3.7	21.5 ± 4.2	0.250	22.3 ± 4.3	22.3 ± 4.7	0.960
Systolic blood pressure, mmHg	113 ± 18	128 ± 18	<0.001	111 ± 17	126 ± 19	<0.001	115 ± 18	131 ± 17	<0.001
Diastolic blood pressure, mmHg	56 ± 6	75 ± 8	<0.001	56 ± 6	75 ± 8	<0.001	56 ± 6	75 ± 9	<0.001
Heart rate, bpm	68 ± 14	72 ± 15	<0.001	70 ± 16	74 ± 15	0.007	67 ± 12	70 ± 15	0.041
Albumin, g/dL	3.4 ± 0.5	3.4 ± 0.4	0.366	3.4 ± 0.5	3.4 ± 0.4	0.968	3.3 ± 0.4	3.4 ± 0.5	0.232
CRP, mg/dL	0.89 ± 1.66	0.67 ± 1.22	0.065	0.66 ± 1.09	0.73 ± 1.41	0.616	1.14 ± 2.08	0.60 ± 0.93	0.005
eGFR, mL/min/1.73 m^2^	43.2 ± 18.6	45.6 ± 19.6	0.234	48.0 ± 18.0	46.8 ± 18.1	0.552	38.0 ± 17.8	43.2 ± 21.1	0.027
log (NT-proBNP)	3.05 ± 0.51	2.99 ± 0.50	0.149	2.96 ± 0.44	2.94 ± 0.47	0.662	3.15 ± 0.55	3.05 ± 0.53	0.159
Atrial fibrillation, *n* (%)	131 (44)	158 (51)	0.072	86 (56)	86 (51)	0.217	45 (32)	72 (50)	0.001
Diabetes mellitus, *n* (%)	93 (32)	91 (30)	0.293	47 (31)	47 (29)	0.339	46 (33)	44 (31)	0.417
Dyslipidemia, *n* (%)	103 (35)	119 (39)	0.229	42 (27)	60 (36)	0.071	61 (43)	59 (42)	0.412
Hypertension, *n* (%)	245 (83)	267 (86)	0.228	119 (77)	138 (83)	0.201	126 (89)	129 (90)	0.484
Medications									
Beta blockers, *n* (%)	149 (51)	166 (53)	0.279	76 (49)	85 (51)	0.476	73 (52)	81 (57)	0.241
Calcium channel blockers, *n* (%)	152 (52)	152 (49)	0.271	63 (41)	68 (40)	0.496	89 (63)	84 (59)	0.262
Diuretics, *n* (%)	243 (82)	255 (82)	0.460	124 (81)	143 (85)	0.204	119 (84)	112 (73)	0.122
RAAS inhibitors, *n* (%)	215 (73)	220 (71)	0.288	115 (75)	119 (70)	0.232	100 (71)	101 (71)	0.530
Statins, *n* (%)	89 (30)	79 (25)	0.106	37 (24)	35 (21)	0.281	52 (37)	44 (31)	0.167

Values are mean ± standard deviation or number (%). CRP, C-reactive protein; eGFR, estimated glomerular filtration rate; LVH, left ventricular hypertrophy; NT-proBNP, N-terminal pro-brain natriuretic peptide; RAAS, renin–angiotensin–aldosterone system.

**Table 4:** Differences in Ed/Ea, an index of echocardiographic diastolic function, before discharge between patients with and without all-cause mortality, in those with and without LVH.

		All-Cause Mortality		*p*-Value (− vs. +)	Diastolic Blood Pressure		*p*-Value (Low vs. High)
	Ed/Ea	−	+		Low	High	
All	0.127 ± 0.057	0.122 ± 0.055	0.146 ± 0.061	<0.001	0.135 ± 0.063	0.119 ± 0.050	<0.001
							
							
LVH−	0.119 ± 0.052	0.115 ± 0.052	0.132 ± 0.052	0.021	0.128 ± 0.061	0.110 ± 0.042	0.003
LVH+	0.136 ± 0.061	0.130 ± 0.058	0.161 ± 0.067	<0.001	0.144 ± 0.064	0.128 ± 0.057	0.033
*p*-value (LVH− vs. +)	<0.001	0.004	0.010		0.027	0.001	

Values are mean ± standard deviation. Ea, arterial elastance; Ed, diastolic elastance; LVH, left ventricular hypertrophy. Ed/Ea = (E/e’)/(0.9 × systolic blood pressure).

**Table 5:** Analytical data of prognostic factors for all-cause mortality in patients with and without left ventricular hypertrophy.

	(A) Left Ventricular Hypertrophy −					
	Cox Hazard Analysis					
	Univariable			Multivariable		
	Ratio	95% CI	*p*-Value	Ratio	95% CI	*p*-Value
Age	1.068	1.034–1.104	<0.001	1.056	1.016–1.096	0.005
Male	1.029	0.63–1.618	0.909	1.621	0.937–2.806	0.084
SBP	0.989	0.975–1.003	0.115	-	-	-
DBP	0.998	0.978–1.02	0.907	-	-	-
Albumin	0.218	0.153–0.516	<0.001	0.396	0.199–0.785	0.008
NT-proBNP	4.757	2.615–8.652	<0.001	2.658	1.358–5.203	0.004
LAVI	1.008	0.999–1.018	0.073	-	-	-
LVMI	0.996	0.981–1.011	0.604	-	-	-
E/e’	1.032	0.990–1.076	0.134	-	-	-
Ed/Ea	38.32	1.277–1149	0.035	91.420	1.213–6893	0.040
	**(B) Left Ventricular Hypertrophy +**					
Age	1.145	1.101–1.191	<0.001	1.145	1.096–1.197	<0.001
Male	1.112	0.636–1.942	0.709	1.306	0.697–2.445	0.404
SBP	0.986	0.972–1.001	0.060	-	-	-
DBP	0.961	0.939–0.984	0.001	0.996	0.968–1.024	0.787
Albumin	0.420	0.261–0.676	<0.001	0.460	0.266–0.795	0.005
NT-proBNP	2.173	1.358–3.477	0.001	1.884	1.054–3.366	0.032
LAVI	1.003	0.996–1.009	0.412	-	-	-
LVMI	0.999	0.990–1.008	0.857	-	-	-
E/e’	1.053	1.017–1.089	0.003	-	-	-
Ed/Ea	1501	48.37–46600	<0.001	142.6	1.612–12610	0.030

DBP, diastolic blood pressure; Ea, arterial elastance; Ed, diastolic elastance; LAVI, left atrial volume index; LVMI, left ventricular mass index; NT-proBNP, N-terminal pro-brain natriuretic peptide; SBP, systolic blood pressure.

## Data Availability

Data set used in this study is available when ordered from corresponding author.

## Supplementary Materials

The following supporting information can be downloaded at: https://www.mdpi.com/article/10.3390/jcm13195911/s1, Table S1: Differences in echocardiographic data before discharge between patients with low and high diastolic blood pressure in those with and without LVH; Table S2: Differences in patient characteristics before discharge between patients with and without all-cause mortality in those with or without LVH; Table S3: Differences in echocardiographic data before discharge between patients with and without all-cause mortality in those with or without LVH.

## Appendix A

The OCVC-Heart Failure Investigators

Masahiro Seo, Tetsuya Watanabe, and Takahisa Yamada, Osaka General Medical Center, Osaka, Japan; Takaharu Hayashi and Yoshiharu Higuchi, Osaka Police Hospital, Osaka, Japan; Masaharu Masuda, Mitsutoshi Asai, and Toshiaki Mano, Kansai Rosai Hospital, Amagasaki, Japan; Hisakazu Fuji, Kobe Ekisaikai Hospital, Kobe, Japan; Shunsuke Tamaki, Daisaku Masuda, Ryu Shutta, and Shizuya Yamashita, Rinku General Medical Center, Izumisano, Japan; Masami Sairyo and Yusuke Nakagawa, Kawanichi City Medical Center, Kawanishi, Japan; Haruhiko Abe, Yasunori Ueda, and Yasushi Matsumura, National Hospital Organization Osaka National Hospital, Osaka, Japan; Kunihiko Nagai, Ikeda Municipal Hospital, Ikeda, Japan; Masamichi Yano, Masami Nishino, and Jun Tanouchi, Osaka Rosai Hospital, Sakai, Japan; Yoh Arita and, Nobuyuki Ogasawara, Japan Community Health Care Organization Osaka Hospital, Osaka, Japan; Takamaru Ishizu, Minoru Ichikawa and Yuzuru Takano, Higashiosaka City Medical Center, Higashiosaka, Japan; Eisai Rin, Kawachi General Hospital, Higashiosaka, Japan; Yukinori Shinoda, Koichi Tachibana and Shiro Hoshida, Yao Municipal Hospital, Yao, Japan; Masahiro Izumi, Kinki Central Hospital, Itami, Japan; Hiroyoshi Yamamoto and Hiroyasu Kato, Japan Community Health Care Organization, Osaka Minato Central Hospital, Osaka, Japan; Kazuhiro Nakatani and Yuji Yasuga, Sumitomo Hospital, Osaka, Japan; Mayu Nishio and Keiji Hirooka, Saiseikai Senri Hospital, Suita, Japan; Takahiro Yoshimura, Kazunori Kashiwase and Shinji Hasegawa, National Hospital Organization Osaka Minami Medical Center, Kawachinagano, Japan; Akihiro Tani, Kano General Hospital, Osaka, Japan; Yasushi Okumoto, Kinan Hospital, Tanabe, Japan; Yasunaka Makino, Hyogo Prefectural Nishinomiya Hospital, Nishinomiya, Japan; Toshinari Onishi and Katsuomi Iwakura, Sakurabashi Watanabe Hospital, Osaka, Japan; Yoshiyuki Kijima, Japan Community Health Care Organization, Hoshigaoka Medical Center, Hirakata, Japan; Takashi Kitao, Minoh City Hospital, Minoh, Japan; Masashi Fujita, Osaka International Cancer Institute, Osaka, Japan; Koichiro Harada, Suita Municipal Hospital, Suita, Japan; Masahiro Kumada and Osamu Nakagawa, Toyonaka Municipal Hospital, Toyonaka, Japan; Ryo Araki and Takayuki Yamada, Otemae Hospital, Osaka, Japan; Akito Nakagawa and Yoshio Yasumura, Amagasaki Chuo Hospital, Amagasaki, Japan; and Yuki Matsuoka, Taiki Sato, Akihiro Sunaga, Bolrathanak Oeun, Hirota Kida, Yohei Sotomi, Tomoharu Dohi, Yasuhiro Akazawa, Kei Nakamoto, Katsuki Okada, Fusako Sera, Hidetaka Kioka, Tomohito Ohtani, Toshihiro Takeda, Daisaku Nakatani, Hiroya Mizuno, Shungo Hikoso, and Yasushi Sakata, Osaka University Graduate School of Medicine, Suita, Japan.

## References

[1] Vrints C., Andreotti F., Koskinas K.C., Rossello X., Adamo M., Ainslie J., Banning A.P., Budaj A., Buechel R.R., Chiariello G.A. (2024). 2024 ESC Guidelines for the Management of Chronic Coronary Syndromes. Eur. Heart J..

[2] Del Buono M.G., Montone R.A., Camilli M., Carbone S., Narula J., Lavie C.J., Niccoli G., Crea F. (2021). Coronary microvascular dysfunction across the spectrum of cardiovascular diseases: JACC state-of-the-art review. J. Am. Coll. Cardiol..

[3] Vancheri F., Longo G., Vancheri S., Henein M. (2020). Coronary microvascular dysfunction. J. Clin. Med..

[4] Dryer K., Gajjar M., Narang N., Lee M., Paul J., Shah A.P., Nathan S., Butler J., Davidson C.J., Fearon W.F. (2018). Coronary microvascular dysfunction in patients with heart failure with preserved ejection fraction. Am. J. Physiol. Heart Circ. Physiol..

[5] Shah S.J., Lam C.S.P., Svedlund S., Saraste A., Hage C., Tan R.S., Beussink-Nelson L., Faxén U.L., Fermer M.L., Broberg M.A. (2018). Prevalence and correlates of coronary microvascular dysfunction in heart failure with preserved ejection fraction: PROMIS-HFpEF. Eur. Heart J..

[6] Yang J.H., Obokata M., Reddy Y.N.V., Redfield M.M., Lerman A., Borlaug B.A. (2020). Endothelium-dependent and independent coronary microvascular dysfunction in patients with heart failure with preserved ejection fraction. Eur. J. Heart Fail..

[7] Ahmad A., Corban M.T., Toya T., Verbrugge F.H., Sara J.D., Lerman L.O., Borlaug B.A., Lerman A. (2021). Coronary microvascular dysfunction is associated with exertional haemodynamic abnormalities in patients with heart failure with preserved ejection fraction. Eur. J. Heart Fail..

[8] Hage C., Svedlund S., Saraste A., Faxén U.L., Benson L., Fermer M.L., Gan L.-M., Shah S.J., Lam C.S.P., Lund L.H. (2020). Association of coronary microvascular dysfunction with heart failure hospitalizations and mortality in heart failure with preserved ejection fraction: A follow-up in the PROMIS-HFpEF study. J. Card. Fail..

[9] Hoshida S., Watanabe T., Shinoda Y., Minamisaka T., Fukuoka H., Inui H., Ueno K., Yamada T., Uematsu M., Yasumura Y. (2020). Considerable scatter in the relationship between left atrial volume and pressure in heart failure with preserved left ventricular ejection fraction. Sci. Rep..

[10] Schindler T.H., Schelbert H.R., Quercioli A., Dilsizian V. (2010). Cardiac PET imaging for the detection and monitoring of coronary artery disease and microvascular health. JACC Cardiovasc. Imaging.

[11] Kotecha T., Martinez-Naharro A., Boldrini M., Knight D., Hawkins P., Kalra S., Patel D., Coghlan G., Moon J., Plein S. (2019). Automated pixel-wise quantitative myocardial perfusion mapping by CMR to detect obstructive coronary artery disease and coronary microvascular dysfunction: Validation against invasive coronary physiology. JACC Cardiovasc. Imaging.

[12] Hoshida S., Shinoda Y., Ikeoka K., Fukuoka H., Inui H., Watanabe T. (2016). Age- and sex-related differences in diastolic function and cardiac dimensions in a hypertensive population. ESC Heart Fail..

[13] Hoshida S. (2023). Due diligence of a diastolic index as a prognostic factor in heart failure with preserved ejection fraction. J. Clin. Med..

[14] Hoshida S. (2023). Left-side pressure index for all-cause mortality in older adults with HFpEF: Diagnostic potential for HFpEF and possible view for HFrEF. J. Clin. Med..

[15] Suna S., Hikoso S., Yamada T., Uematsu M., Yasumura Y., Nakagawa A., Takeda T., Kojima T., Kida H., Oeun B. (2020). Study protocol for the PURSUIT-HFpEF study: A prospective, multicenter, observational study of patients with heart failure with preserved ejection fraction. BMJ Open.

[16] Matsumura Y., Hattori A., Manabe S., Takahashi D., Yamamoto Y., Murata T., Nakagawa A., Mihara N., Takeda T. (2017). Case report form reporter: A key component for the integration of electronic medical records and the electronic data capture system. Stud. Health Technol. Inform..

[17] Nagueh S.F., Smiseth O.A., Appleton C.P., Byrd B.F., Dokainish H., Edvardsen T., Flachskampf F.A., Gillebert T.C., Klein A.L., Lancellotti P. (2016). Recommendations for the evaluation of left ventricular diastolic function by echocardiography: An update from the American Society of Echocardiography and the European Association of Cardiovascular Imaging. J. Am. Soc. Echocardiogr..

[18] Lang R.M., Badano L.P., Mor-Avi V., Afilalo J., Armstrong A., Ernande L., Flachskampf F.A., Foster E., Goldstein S.A., Kuznetsova T. (2015). Recommendations for cardiac chamber quantification by echocardiography in adults: An update from the American Society of Echocardiography and the European Association of Cardiovascular Imaging. J. Am. Soc. Echocardiogr..

[19] Cecchi F., Olivotto I., Gistri R., Lorenzoni R., Chiriatti G., Camici P.G. (2003). Coronary microvascular dysfunction and prognosis in hypertrophic cardiomyopathy. N. Engl. J. Med..

[20] Camici P.G., Tschöpe C., Di Carli M.F., Rimoldi O., Van Linthout S. (2020). Coronary microvascular dysfunction in hypertrophy and heart failure. Cardiovasc. Res..

[21] Taqueti V.R., Solomon S.D., Shah A.M., Desai A.S., Groarke J.D., Osborne M.T., Hainer J., Bibbo C.F., Dorbala S., Blankstein R. (2018). Coronary microvascular dysfunction and future risk of heart failure with preserved ejection fraction. Eur. Heart J..

[22] Adingupu D.D., Göpel S.O., Grönros J., Behrendt M., Sotak M., Miliotis T., Dahlqvist U., Gan L.-M., Jönsson-Rylander A.-C. (2019). SGLT2 inhibition with empagliflozin improves coronary microvascular function and cardiac contractility in prediabetic ob/ob^−/−^ mice. Cardiovasc. Diabetol..

[23] Juni R.P., Kuster D.W.D., Goebel M., Helmes M., Musters R.J.P., van der Velden J., Koolwijk P., Paulus W.J., van Hinsbergh V.W. (2019). Cardiac microvascular endothelial enhancement of cardiomyocyte function is impaired by inflammation and restored by empagliflozin. JACC Basic Transl. Sci..

[24] Mazer C.D., Hare G.M.T., Connelly P.W., Gilbert R.E., Shehata N., Quan A., Teoh H., Leiter L.A., Zinman B., Jüni P. (2020). Effect of empagliflozin on erythropoietin levels, iron stores, and red blood cell morphology in patients with type 2 diabetes mellitus and coronary artery disease. Circulation.

[25] Kanbay M., Tapoi L., Ureche C., Tanriover C., Cevik E., Demiray A., Afsar B., Cherney D.Z.I., Covic A. (2022). Effect of sodium-glucose cotransporter 2 inhibitors on hemoglobin and hematocrit levels in type 2 diabetes: A systematic review and meta-analysis. Int. Urol. Nephrol..

[26] Li M., Yi T., Fan F., Qiu L., Wang Z., Weng H., Ma W., Zhang Y., Huo Y. (2022). Effect of sodium-glucose cotransporter-2 inhibitors on blood pressure in patients with heart failure: A systematic review and meta-analysis. Cardiovasc. Diabetol..

[27] Patel S.M., Kang Y.M., Im K., Neuen B.L., Anker S.D., Bhatt D.L., Butler J., Cherney D.Z., Claggett B.L., Fletcher R.A. (2024). Sodium-Glucose Cotransporter-2 Inhibitors and Major Adverse Cardiovascular Outcomes: A SMART-C Collaborative Meta-Analysis. Circulation.

