# Supplementary material for: Alternative Factors in Possible Involvement of Coronary Microvascular Dysfunction in Older Patients with HFpEF
Source: J Clin Med. 2024 Oct 3;13(19):5911. doi: 10.3390/jcm13195911 (PMC11477810; doi:10.3390/jcm13195911)
Supplement: Supplementary file 1 [file jcm-13-05911-s001.zip › Supplementary Table S1.pdf]

Suppl. Table S1. Differences in echocardiographic data before discharge between patients with low and high diastolic blood pressure in those with and without LVH

	Diastolic blood pressure		*p- value*	LVH - Diastolic blood pressure		*p- value*	LVH + Diastolic blood pressure		*p- value*
	**Low**	**High**	*(- vs +)*	**Low**	**High**	*(- vs +)*	**Low**	**High**	*(- vs +)*
**LAVI, mL/m²**	**54.6 ± 25.5**	**55.6 ± 34.8**	***0.688***	**51.0 ± 23.6**	**48.9 ± 25.7**	***0.460***	**58.6 ± 27.0**	**63.6 ± 41.9**	***0.268***
**LVEDVI, mL/m²**	**55.6 ± 21.0**	**54.2 ± 20.2**	***0.426***	**50.1 ± 17.4**	**49.8 ± 18.4**	***0.896***	**61.2 ± 22.8**	**59.1 ± 21.1**	***0.436***
**LVESVI, mL/m²**	**21.8 ± 10.6**	**22.0 ± 10.9**	***0.858***	**19.8 ± 9.3**	**20.0 ± 9.7**	***0.917***	**23.8 ± 11.5**	**24.2 ± 11.6**	***0.779***
**LVEF, %**	**61.1 ± 7.7**	**60.5 ± 7.7**	***0.365***	**61.1 ± 8.3**	**60.7 ± 7.4**	***0.705***	**61.2 ± 6.8**	**60.3 ± 7.9**	***0.346***
**LVMI, g/m²**	**108.2 ± 35.3**	**104.1 ± 33.1**	***0.148***	**84.6 ± 14.1**	**81.9 ± 16.6**	***0.137***	**133.9 ± 32.5**	**130.5 ± 27.9**	***0.341***
**TRPG, mmHg**	**28.9 ± 9.8**	**27.8 ± 9.1**	***0.156***	**28.6 ± 9.9**	**27.6 ± 8.5**	***0.352***	**29.3 ± 9.6**	**28.0 ± 9.8**	***0.295***
**E/A**	**1.0 ± 0.6**	**1.0 ± 0.7**	***0.642***	**1.0 ± 0.6**	**1.0 ± 0.7**	***0.894***	**1.0 ± 0.5**	**1.0 ± 0.5**	***0.530***
**DcT of E wave**	**0.21 ± 0.06**	**0.21 ± 0.06**	***0.315***	**0.21 ± 0.06**	**0.21 ± 0.06**	***0.666***	**0.22 ± 0.06**	**0.21 ± 0.05**	***0.068***
**E/e´**	**13.5 ± 5.9**	**13.5 ± 5.6**	***0.915***	**12.5 ± 5.4**	**12.3 ± 4.6**	***0.788***	**14.5 ± 6.1**	**14.9 ± 6.3**	***0.617***

Values are mean ± standard deviation.

LAVI, left atrial volume index;
LVEDVI, left ventricular end-diastolic volume index; LVESVI, left ventricular end-systolic volume index
LVEF, left ventricular ejection fraction; LVH, left ventricular hypertrophy;
LVMI, left ventricular mass index; TRPG, tricuspid regurgitation pressure gradient;
DcT, deceleration time; E, early transmitral flow velocity; e', onset of early diastolic mitral annular velocity; Ed diastolic elastance; Ea, arterial elastance.
