# Supplementary material for: Alternative Factors in Possible Involvement of Coronary Microvascular Dysfunction in Older Patients with HFpEF
Source: J Clin Med. 2024 Oct 3;13(19):5911. doi: 10.3390/jcm13195911 (PMC11477810; doi:10.3390/jcm13195911)
Supplement: Supplementary file 1 [file jcm-13-05911-s001.zip › Supplementary Table S2.pdf]

Suppl. Table S2. Differences in patient characteristics before discharge between patients with and without all-cause mortality in those with and without LVH

	LVH - (n=323)			LVH + (n=284)		
	All-cause mortality		*p-value*	All-cause mortality		*p-value*
	- (n = 259)	+ (n = 64)	*(- vs. +)*	- (n = 225)	+ (n = 59)	*(- vs. +)*
Age, years	80.6 ± 9.3	84.9 ± 7.6	*<0.001*	79.3 ± 9.6	87.0 ± 6.5	*<0.001*
Male, n (%)	127 (49)	33 (52)	*0.717*	72 (32)	18 (31)	*0.826*
Body mass index, Kg/m^2	21.7 ± 4.0	19.8 ± 3.6	*<0.001*	22.5 ± 4.5	21.6 ± 4.3	*0.174*
Systolic blood pressure, mmHg	120 ± 19	115 ± 21	*0.076*	124 ± 19	120 ± 20	*0.129*
Diastolic blood pressure, mmHg	66 ± 12	65 ± 12	*0.631*	67 ± 12	61 ± 9	*0.001*
Heart rate, bpm	73 ± 16	71 ± 15	*0.431*	68 ± 13	71 ± 16	*0.110*
Albumin, g/dL	3.5 ± 0.4	3.2 ± 0.5	*<0.001*	3.4 ± 0.4	3.2 ± 0.4	*<0.001*
CRP, mg/dL	0.69 ± 1.30	0.71 ± 1.12	*0.929*	0.80 ± 1.64	1.17 ± 1.59	*0.125*
eGFR, mL/min/1.73m^2	48.1 ± 17.3	44.6 ± 20.5	*0.167*	41.1 ± 18.8	38.5 ± 23.1	*0.364*
log (NT-proBNP)	2.88 ± 0.44	3.21 ± 0.42	*<0.001*	3.05 ± 0.54	3.29 ± 0.50	*0.004*
Atrial fibrillation, n (%)	137 (53)	35 (55)	*0.797*	96 (43)	21 (36)	*0.325*
Diabetes mellitus, n (%)	76 (30)	18 (29)	*0.847*	68 (30)	22 (37)	*0.229*
Dyslipidemia, n (%)	88 (34)	14 (23)	*0.062*	101 (45)	19 (32)	*0.079*
Hypertension, n (%)	209 (81)	48 (75)	*0.311*	203 (90)	52 (88)	*0.637*
Medications						
Beta-blockers, n (%)	128 (50)	33 (52)	*0.759*	128 (57)	26 (44)	*0.078*
Calcium-channel blockers, n	103 (40)	28 (44)	*0.561*	144 (64)	29 (49)	*0.037*
Diuretics, n (%)	213 (82)	54 (84)	*0.686*	177 (79)	54 (92)	*0.038*
RAAS inhibitors, n (%)	188 (73)	46 (72)	*0.909*	164 (73)	37 (63)	*0.126*
Statins, n (%)	60 (23)	12 (19)	*0.447*	78 (35)	18 (31)	*0.547*

Values are mean ± standard deviation or number (%).
CRP, C-reactive protein; eGFR, estimated glomerular filtration rate;
LVH, left ventricular hypertrophy; NT-proBNP, N-terminal pro-brain natriuretic peptide;
RAAS, renin-angiotensin-aldosterone system
