# Supplementary material for: Alternative Factors in Possible Involvement of Coronary Microvascular Dysfunction in Older Patients with HFpEF
Source: J Clin Med. 2024 Oct 3;13(19):5911. doi: 10.3390/jcm13195911 (PMC11477810; doi:10.3390/jcm13195911)
Supplement: Supplementary file 1 [file jcm-13-05911-s001.zip › Supplementary Table S3.pdf]

**Suppl. Table S3. Differences in echocardiographic data before discharge between patients with and without all-cause mortality in those with and without LVH**

| | LVH - | | | LVH + | | |
|---|---|---|---|---|---|---|
| | All-cause mortality | | *p- value* | All-cause mortality | | *p- value* |
| | - | + | *(- vs +)* | - | + | *(- vs +)* |
| LAVI, mL/m$^2$ | 48.8 ± 23.5 | 54.2 ± 28.5 | *0.129* | 60.3 ± 37.3 | 64.9 ± 27.2 | *0.423* |
| LVEDVI, mL/m$^2$ | 50.1 ± 18.3 | 49.6 ± 16.2 | *0.856* | 61.0 ± 22.7 | 57.1 ± 18.6 | *0.247* |
| LVESVI, mL/m$^2$ | 19.9 ± 9.7 | 20.0 ± 8.3 | *0.912* | 24.5 ± 12.1 | 22.3 ± 8.8 | *0.219* |
| LVEF, % | 61.2 ± 8.1 | 59.5 ± 6.8 | *0.123* | 60.6 ± 7.6 | 61.4 ± 6.4 | *0.473* |
| LVMI, g/m$^2$ | 83.3 ± 16.4 | 82.6 ± 16.5 | *0.775* | 132.7 ± 31.4 | 130.0 ± 25.8 | *0.531* |
| TRPG, mmHg | 27.7 ± 9.1 | 29.6 ± 9.5 | *0.158* | 27.8 ± 9.0 | 31.7 ± 11.6 | *0.006* |
| E/A | 1.0 ± 0.7 | 1.0 ± 0.6 | *0.843* | 1.0 ± 0.5 | 0.9 ± 0.4 | *0.552* |
| DcT of E wave | 0.21 ± 0.06 | 0.21 ± 0.06 | *0.828* | 0.21 ± 0.05 | 0.22 ± 0.05 | *0.193* |
| E/e´ | 12.2 ± 5.0 | 13.3 ± 4.8 | *0.128* | 14.2 ± 6.2 | 16.6 ± 5.9 | *0.008* |

**Values are mean ± standard deviation.**

**LAVI, left atrial volume index;**
**LVEDVI, left ventricular end-diastolic volume index; LVESVI, left ventricular end-systolic volume index; LVEF, left ventricular ejection fraction; LVH, left ventricular hypertrophy;**
**LVMI, left ventricular mass index; TRPG, tricuspid regurgitation pressure gradient;**
**DcT, deceleration time; E, early transmitral flow velocity; e', onset of early diastolic mitral annular velocity**
